# Virtual Monoenergetic Imaging of Thoracoabdominal Computed Tomography Angiography on Photon-Counting Detector Computertomography: Assessment of Image Quality and Leveraging Low-keV Series for Salvaging Suboptimal Contrast Acquisitions

**DOI:** 10.3390/diagnostics14242843

**Published:** 2024-12-17

**Authors:** Katharina Rippel, Josua A. Decker, Jan Luitjens, Osama Habeeballah, Stefanie Bette, Franziska Braun, Thomas J. Kroencke, Christian Scheurig-Muenkler

**Affiliations:** 1 Diagnostic and Interventional Radiology, University Hospital Augsburg, Faculty of Medicine, University of Augsburg, Stenglinstr. 2, 86156 Augsburg, Germany; 2 Centre for Advanced Analytics and Predictive Sciences (CAAPS), University of Augsburg, Universitätsstr. 2, 86159 Augsburg, Germany

**Keywords:** photon-counter detector CT, high-pitch ECG-gated aortic CT angiographies, virtual monoenergetic imaging, low-contrast attenuation scans

## Abstract

Background: The aim of this study was to assess the possibility of image improvement of ECG-gated, high-pitch computed tomography angiography (CTA) of the thoracoabdominal aorta before transaortic valve replacement (TAVR) on a novel dual-source photon-counting detector CT (PCD-CT) in the setting of suboptimal low-contrast attenuation. Methods: Continuously examined patients who underwent an ECG-gated, high-pitch CTA of the aorta on a PCD-CT with a contrast decrease of at least 50% between the ascending aorta and the common femoral arteries (CFA) were included. Patient characteristics were documented. Virtual monoenergetic imaging (VMI) reconstructions with three keV settings were generated. CT values and noise were measured for five vascular segments of the aorta and the CFA. Signal-to-noise (SNR) and contrast-to-noise ratios (CNR) were calculated. Two independent board-certified radiologists rated the images with the focus on vascular attenuation, vessel sharpness, and image quality using a 5-point Likert scale. Results: Fifty-five patients (mean age 77.4 ± 8.5 years; 15 women) were included. The SNR was significantly higher at 40 and 45 keV VMI compared to reference 70 keV (*p* < 0.001 and *p* = 0.005, respectively). The same was shown for the CNR (*p* < 0.001 and *p* = 0.0049, respectively). Subjective image evaluation showed a significant increase in vessel attenuation in the lower keV reconstructions, while the overall image quality decreased only slightly. Furthermore, 50% (8/16) of primarily non-diagnostic scans were considered diagnostic when using low-keV reconstructions (*p* > 0.05). Conclusions: ECG-gated CTA of the aorta in high-pitch mode on PCD-CT with suboptimal contrast enhancement at the level of the CFA can be salvaged by using low-keV VMI. This implies the possibility of radiation dose reduction by eliminating the need for repeat scans.

## 1. Introduction

The advantages of photon-counting detector CT (PCD-CT) acquisition have been proven in various studies [1,2,3]. Besides the elimination of electronic background noise from image acquisition, all scans include spectral information from which virtual monoenergetic images (VMIs) can be reconstructed [4,5]. As seen in several studies on dual-energy energy-integrating detector CT, reconstructing low-keV VMIs offers the possibility of reducing the iodine load to about 50% while maintaining the radiation dose [6,7]. Recent studies have shown that this principle can also be applied to PCD-CT [8,9]. The decrease in noise levels due to the use of PCD-CT in low-keV reconstructions offers new possibilities for diagnostic improvement. There are several studies that propose a potential reduction in the contrast media concentration of up to 50% when scanning the thoracoabdominal aorta or the coronary arteries [10,11]. However, another aspect is the potential to increase diagnostic confidence in CT angiographies with low attenuation to circumvent a repetition and hence an additional application of the contrast agent and radiation in patients with non-diagnostic images [12].

Transcatheter aortic valve replacement (TAVR) is a standard procedure for the treatment of aortic valve stenosis nowadays [13]. For planning purposes, a computed tomography angiography of the thoracoabdominal aorta is required [14]. Typically, a high-pitch computed tomography angiography (CTA) with ECG gating is performed to evaluate cardiac morphology and femoral access sites [14,15,16]. However, 5–10% of the patients being evaluated for TAVR suffer from low-flow, low-gradient, severe aortic stenosis. About half of them have a highly reduced left ventricular ejection fraction [17]. This explains why a certain number of CT scans show suboptimal contrast attenuation at the level of the common femoral arteries. The potential benefits of PCD-CT acquisition during pre-TAVR workup have been shown by various studies [18,19,20]. However, it remains unclear whether the inherent spectral information can be utilized to salvage scans with suboptimal contrast of the femoral access route.

This study analyzes the objective and subjective image quality of CT angiographies of the thoracoabdominal aorta in cases of suboptimal contrast attenuation at the level of the femoral arteries with ECG gating and a high-pitch factor performed at an PCD. This was done to analyze the potential for image improvement in the setting of suboptimal or even non-diagnostic scans of the access site in pre-TAVR CTA.

## 2. Materials and Methods

The Medical Research and Ethics Committee of the Ludwig-Maximilians-University approved the protocol of this retrospective subgroup evaluation of prospectively collected study data, and informed consent was obtained from all participants. The ethical standards of the 1946 Declaration of Helsinki and its later amendments were adhered to during the conduction of this study.

### 2.1. Patient Population

All ECG-gated, high-pitch CTA of the thoracoabdominal aorta before TAVR that were acquired between August 2021 and May 2023 on a dual-source photon-counting detector CT (NAEOTOM alpha, Siemens Healthcare GmbH, Forchheim, Germany) were assessed for low-contrast attenuation at the level of the common femoral arteries compared to the ascending aorta. Only scans with an adequate contrast attenuation of the ascending aorta were checked for such decrease in attenuation. This was ensured by using bolus tracking in the ascending aorta during acquisition. Low femoral contrast attenuation was defined as a 50% decrease in attenuation between the ascending aorta and the common femoral artery measured by circular regions of interest (ROIs) in the 70 keV reconstructions. No differentiation was made as to the cause of this low-contrast attenuation. The only exclusion criteria were missing spectral image data, a missing consent form, and an attenuation equal or less than 40 Hounsfield units (HU) in the femoral artery as a sign of no contrast medium. General information such as age, gender, height, and weight were obtained from electronic medical records for all included patients.

### 2.2. Scan Protocol and Image Reconstruction

The monophasic contrast protocol consisted of 120 mL contrast material bolus (Ultravist 300, Bayer Healthcare, Berlin, Germany) followed by 20 mL of a saline chaser. Both were injected via a power injector with a flow of 5 mL/s over an antecubital intravenous canula. First, a scan of the heart was acquired after bolus triggering in the ascending aorta. The used enhancement threshold was set to 100 HU, and the start of the scan was delayed by five seconds and a scan time of 10 s. Immediately afterwards, a second arterial scan stretching from the third cervical vertebra to the groin was started. If the scan was deemed to show a suboptimal contrast of the access vessels by the responsible radiologist, an additional scan of the abdomen was acquired in a late-arterial phase during the same examination. This was performed by visual assessment of the femoral arteries.

All scans were acquired at 120 kVp with an image quality level of 64. Spectral acquisition mode (‘QuantumPlus’, Siemens Healthineers, Forchheim, Germany) using four detector-based energy thresholds (20/30/65/70 keV) was employed. Rotation time, collimation, and pitch were 0.25 s, 144 × 0.4, and 3.2, respectively.

Medium smooth kernels and iterative reconstruction algorithms were used (Bv36 + QIR 3, kernels and algorithms from Siemens Healthineers). Additionally, an enhanced DICOM format (‘Spectral Postprocessing’ = SPP) was chosen that preserved the spectral information. 

The reconstructions were realized in axial orientation with a manually adapted field-of-view with a matrix size of 512. The slice thickness was set to 1.5 mm, and the increment was set to 1 mm. 

Commercially available software (Syngo.via VB70A, Siemens Healthineers) was used to generate VMI reconstructions with identical field-of-view, matrix, and slice positioning at various keV levels (40, 45 and 70 keV). We decided to forego the reconstruction of other keV levels due to the results of preceding studies, in which a clear advantage could be shown for keV level 40 and 45 [12,21].

### 2.3. Objective Image Analysis

A dedicated workstation with open source (ImageJ 1.53c, NIH, Bethesda, MD, USA) and commercially available software (Onis 2.5, Tokyo, Japan) were used for objective image analysis. Circular regions of interest (ROIs) were placed in the ascending, descending, and infrarenal aorta, as well as the common femoral arteries. The measurements in the common femoral arteries were pairwise. ROIs were initially positioned on the 70 keV VMIs and automatically copied to all other keV levels. This guarantees identical voxels and positioning of each dataset only in keV levels.

From all ROIs, mean and standard deviation (SD) of CT values (HU) were deduced to calculate approximations for intravascular contrast (*Signal_ROI_*) and noise (*Noise_ROI_*), respectively. For each ROI, the SNR and CNR were calculated as follows [22]:

$${SNR}_{ROI}=\frac{{Mean\;Signal\;}_{ROI}}{{\;Noise}_{ROI}}$$

$${CNR}_{ROI}=\frac{{(Mean\;Signal}_{ROI}-{Mean\;Signal}_{Psoas})}{{\;Noise}_{ROI}}$$

### 2.4. Subgroup Analysis

Four subgroups were created according to the decrease in the contrast attenuation comparing the common femoral artery to the ascending aorta (subgroup 1: below 20%; subgroup 2: >20–30%, subgroup 3: >30–40%, subgroup 4: >40–50%). Since there were differences in attenuation when looking at the two common femoral arteries of one patient, each singular common femoral artery was individually selected to be assigned to the above-mentioned subgroups. This was done to prevent an otherwise possible impact on the rating.

By using this approach, we were able to distribute 95 measurements in the above defined groups (55 patients × two = 110 femoral arteries). The remaining 15 femoral arteries showed a decrease in attenuation of less than 50% and were thus excluded from the subgroup analysis. Furthermore, the additional scans in the late arterial phase were checked for diagnostic usability in a consensus decision.

### 2.5. Subjective Image Analysis

The subjective image quality was rated independently by two board-certified radiologists (K.R. and C.S.) with 9 and 16 years of experience.

Overall, 165 datasets were evaluated by each reader. To guarantee the blinding of both readers to the reconstruction and postprocessing settings, all datasets were pseudonymized, and all information about the reconstruction parameters was removed. Datasets were presented in random order. This approach guaranteed that readers could not infer the keV levels of the VMIs. Both readers independently rated the overall image quality, the vessel attenuation, and the vessel sharpness on a 5-point Likert scale (1 = non-diagnostic, 2 = poor with restricted diagnostic confidence, 3 = moderate diagnostic confidence, 4 = good diagnostic confidence, and 5 = excellent diagnostic confidence). The definition for a diagnostic usable examination was a rating of 3 or above in all three categories.

### 2.6. Statistical Analysis

Data are presented as mean ± standard deviation or as median with interquartile range (IQR) as indicated. The Shapiro–Wilk test was used to assess normal distribution of data. When comparing two groups with continuous variables, Student’s *t*-test for normally distributed data and the Wilcoxon–Mann–Whitney test for nonparametric data were used. Categorial data were compared using the Chi-square test. To compare multiple groups, the Kruskal–Wallis test with post hoc Bonferroni adjustment was used. Inter-reader agreement was assessed using Krippendorff’s alpha with the following interpretation: 0.0–0.20: slight; 0.21–0.40: fair; 0.41–0.60: moderate; 0.61–0.80: substantial; 0.81–1.0: excellent. Analyses were performed using R version 4.1.0 (R Foundation for Statistical Computing, Vienna, Austria). Statistically significant differences were assumed at *p*-values < 0.05 and at *p*-values < 0.01 for multiple testing.

## 3. Results

### 3.1. Patient Population

During the 22-month period, 687 ECG-gated, high-pitch CTAs of the thoracoabdominal aorta were acquired on the PCD-CT. In 59 cases, the required decrease in contrast attenuation of at least 50% was observed when comparing the common femoral arteries to the ascending aorta at keV level 70, which correlates with the images of an energy-integrating detector acquired with 120 kV, as seen in previous studies [12,21]. Four patients had to be excluded due to following reasons: missing informed consent (*n* = 2), missing spectral data (*n* = 1), and missing contrast enhancement of the common femoral arteries as defined above (*n* = 1). Finally, 55 patients (mean age 77.4 ± 8.5 years; 15 women) were included in the subsequent analyses. Table 1 shows the basic demographic data.

### 3.2. Objective Image Analysis

Table 2 shows the distribution of quantitative CT parameters for different keV levels at the measured localizations.

**Figure 1:**
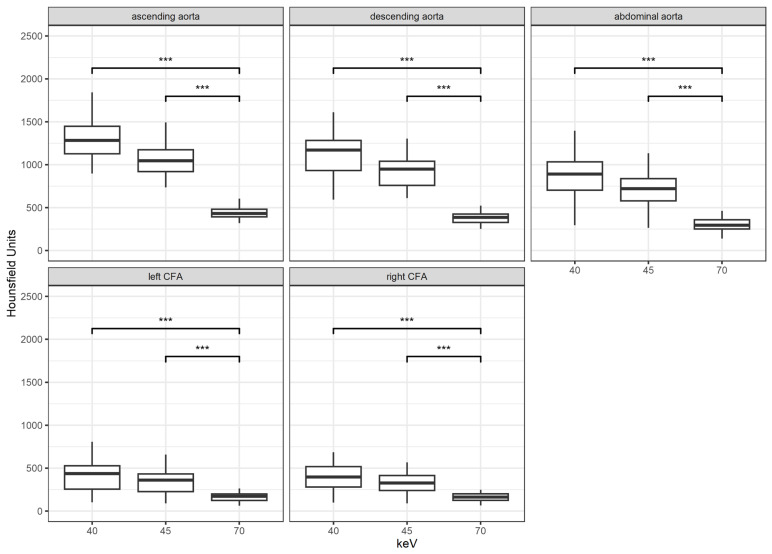
Results for attenuation according to location and keV levels; ***: *p* < 0.001; CFA = common femoral artery.

With each reconstruction step to a lower keV level, the mean attenuation increased. The difference in density is highly significant between the keV levels of 70 vs. 40 and 45 (*p* < 0.001 for both) at any measured location. The increase in density between the VMI with 45 keV to 40 keV was barely significant at the level of the common femoral artery (*p* = 0.048). However, at any other measured location (ascending, descending, and infrarenal aorta), the increase was highly significant (*p* < 0.001). Figure 1 shows the results.

**Figure 2:**
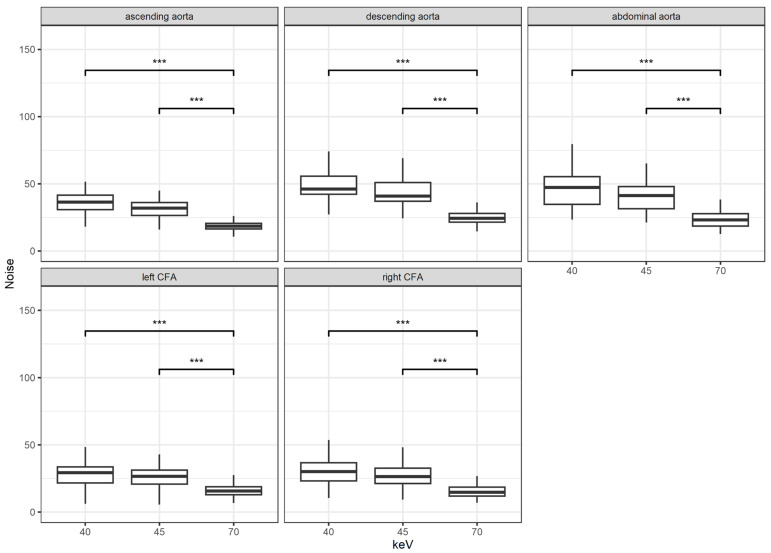
Results for noise according to location and keV levels; ***: *p* < 0.001; CFA = common femoral artery.

The image noise decreased with increasing keV levels and was significantly lower for the keV level of 70 compared to the keV levels 40 and 45 (*p* < 0.001 for both) at all measured sites. When looking at the lower VMI with 40 and 45 keV, the only significant difference could be found at the location of the ascending and descending aorta (*p* < 0.01 and < 0.015, respectively). The results are shown in Figure 2.

**Figure 3:**
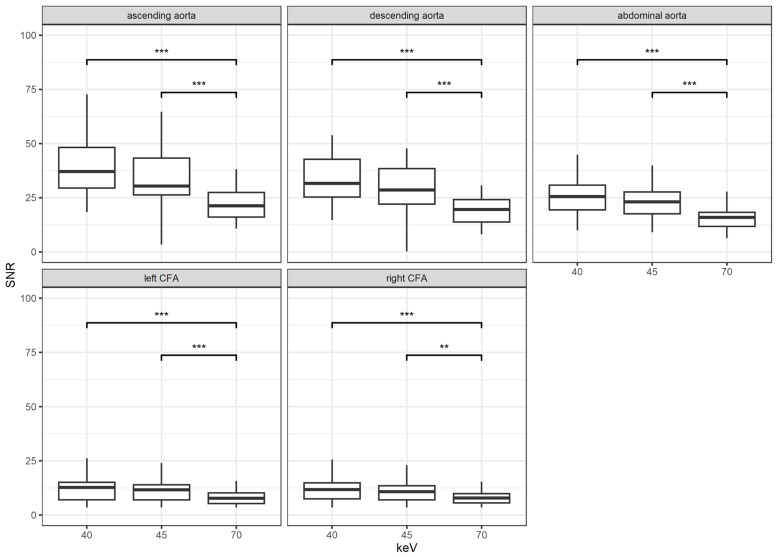
Results for the signal-to-noise ratio (SNR) according to location and keV levels; **: *p* < 0.01; ***: *p* < 0.001; CFA = common femoral artery.

Figure 3 shows the results for the analysis of the SNR. The SNR increased with decreasing keV energy. There was no significant difference between the two lower keV levels of 40 and 45 at any location (all *p* > 0.05). However, there was a significant increase in SNR when comparing the highest keV level of 70 to the two lower levels of 40 and 45 at any measurement site (*p* < 0.001 and *p* = 0.005, respectively).

**Figure 4:**
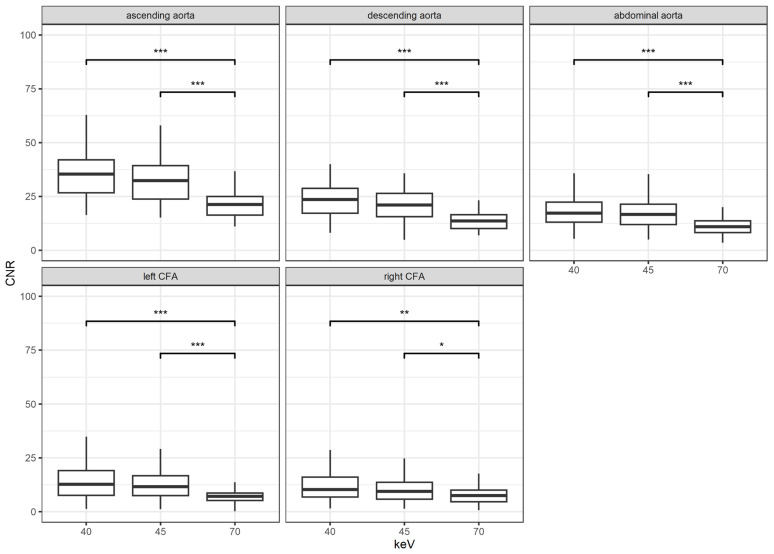
Results for the contrast-to-noise ratio (CNR) according to location and keV levels; *: *p* < 0.05; **: *p* < 0.01; ***: *p* < 0.001; CFA = common femoral artery.

Similarly to SNR, CNR also increased with decreasing keV levels, as shown in Figure 4. Once more, there was no significant difference between the VMI with keV 40 and 45 (all *p* > 0.05). In comparison to the reconstructions with keV level of 70, a highly significant increase in CNR was noticed for the VMI with keV 40 and 45 (*p* < 0.001 and *p* = 0.005, respectively). These results were shown for all measurement sites with an emphasis of significances for the ascending, descending, and infrarenal aorta.

### 3.3. Subgroup Analysis

**Figure 5:**
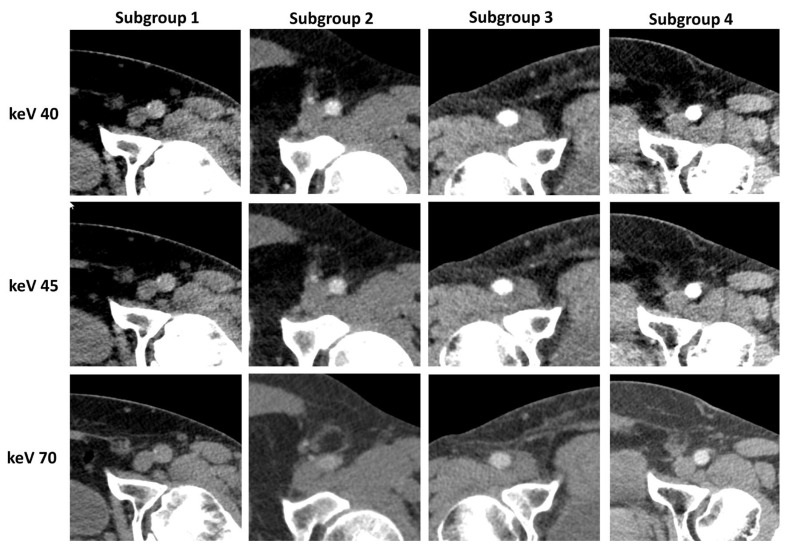
Image examples for each subgroup and keV level.

Of the 95 included CFA, 11 femoral arteries demonstrated a decrease in contrast attenuation below 20% compared with the contrast in the ascending aorta (subgroup 1), 22 demonstrated more than 20% but less than 30% (subgroup 2), and 21 demonstrated more than 30% but less than 40% (subgroup 3). The remaining 41 CFA showed a decrease in attenuation of more than 40 but less than 50% (subgroup 4). Representative reconstructions of all keV levels with an example for each subgroup are shown in Figure 5.

In any of the four groups, the attenuation, noise, CNR, and SNR increased with the decrease in keV levels. This correlates to the overall results measured without the subgroup analysis. This is the reason why we put the focus on the improvement of signal- and contrast-to-noise ratios in the subgroups.

**Figure 6:**
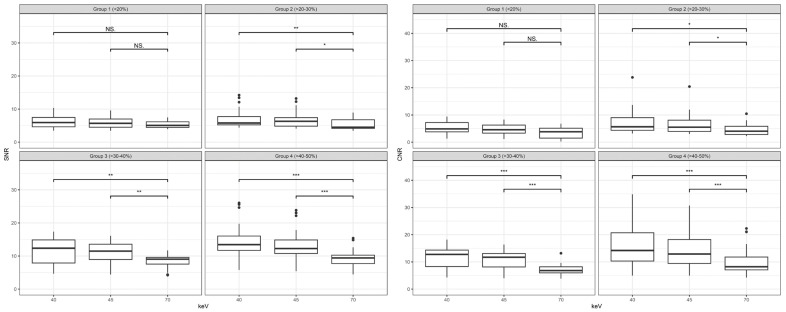
Results for the signal-to-noise ratio (SNR) on the left and contrast-to-noise ratio (CNR) on the right distributed by subgroups and keV levels; NS: not significant *: *p* < 0.05; **: *p* < 0.01; ***: *p* < 0.001.

Figure 6 shows the results of SNR and CNR for all subgroups. Despite the average increase in the attenuation of 102% between keV level 40 and 70, as well as 73% between keV level 45 and 70 in subgroup 1, the increase in CNR and SNR was not significant (*p* > 0.05). However, all the other subgroups showed a significant increase in SNR and CNR when comparing keV level 40 and 45 to the keV level of 70. Especially in the subgroups 3 and 4, the increase was particularly notable for the CNR (*p* < 0.001 for both subgroups) and SNR (*p* < 0.01 and *p* < 0.001, respectively). There was no significant difference between the keV levels of 40 and 45 in all of the subgroups (all *p* > 0.05).

Twenty-seven patients (49%) received an additional scan of the abdomen in the late-arterial phase during the same examination with an average dose length product (DLP) of 122 ± 96.7 mGy·cm. All commune femoral arteries that were in subgroups 1 and 2 and 18 of 21 CFA in subgroup 3 received an additional scan (*n* = 50).

#### Subjective Image Analysis

**Figure 7:**
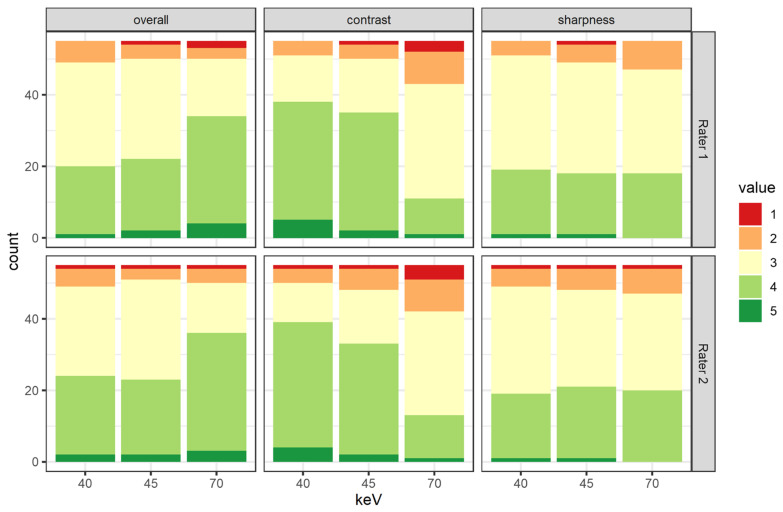
Results of the subjective rating divided by rater, keV level, and rating criteria.

Figure 7 shows the detailed results by rater and category. The interrater agreement was substantial for overall image quality and vessel sharpness (Krippendorff’s α = 0.640 and 0.674, respectively) and moderate for contrast attenuation (Krippendorff’s α = 0.601). There was no significant difference between any assessment of the two raters.

The attenuation of VMI at a keV level of 70 keV was rated significantly lower than any other keV level (2.9 ± 0.8 vs. 3.6 ± 0.7 and 3.7 ± 0.7, respectively, both *p* < 0.001). There was no significant difference between the vessel attenuation of the two lower keV levels (3.7 ± 0.7 for keV level 40 and 3.6 ± 0.7 for keV level 45, *p* = 0.29).

When looking at the overall image quality, the keV level of 70 had significantly higher scores than the VMI with keV level 40 or 45 (3.7 ± 0.7 vs. 3.3 ± 0.7 and 3.4 ± 0.7, respectively, both *p* < 0.01). However, there was no significant difference between the two lower keV levels (*p* = 0.59).

The vessel sharpness was deemed independent from the keV reconstruction level by the raters (*p* = 0.5). All of the additional late arterial phase scans but one were deemed diagnostic. This one non-diagnostic repetition is due to a delayed decision from the responsible radiologist, which meant a scan in the venous phase with insufficient contrast attenuation in the CFA.

When using the above-mentioned division in subgroups and the definition of diagnostic usable scans, the lower keV reconstructions rendered more usable scans. Primarily, the images of 16 patients were deemed non-diagnostic by both readers. Eight of those scans were considered redeemable when using low-keV VMIs (*p* = 0.081). There was no subjective difference in those eight patients when using keV levels of 40 or 45 keV. Table 3 shows the number of nondiagnostic scans divided by subgroups.

## 4. Discussion

This study is the first to present clinical results on the new PCD-CT for thoracoabdominal CTA with suboptimal contrast attenuation in a large patient group. When analyzing the subjective image quality, we were able to show a significant advantage of low-keV VMI reconstruction. This was further confirmed by the objective image analysis in which we were able to demonstrate the possibility to improve image quality in low attenuation and even salvage primarily non-diagnostic scans when using low-keV reconstructions.

Our study showed a significant increase in noise and contrast attenuation in lower keV levels, resulting in a significantly higher CNR at 40 and 45 keV compared to the standard of 70 keV. This is consistent with previous studies [12,21], which also showed that there was an increase in noise and contrast attenuation in PCD-CTs of the thoracoabdominal aorta or the common femoral arteries without special focus on low-contrast attenuation. However, the overall image quality in our study shows a significant decrease in the lower keV levels. This contradiction to Euler et al.’s results may be a result of the special selection of our patients [21]. The overall image quality includes diagnostic certainty, and, due to the patient selection, it was liable that not all scans would be usable for diagnostics. Nevertheless, we were able to increase the number of usable examinations from 39 to 47 (71% to 85%) by using a reconstruction of 40 keV or 45 keV. This means that 50% of the primarily deemed unusable scans were made usable by reconstructing low-keV VMIs (8/16). Furthermore, by using the low-keV reconstruction, additional scans of the abdomen could have been avoided, and thus a reduction in the radiation dose could have been achieved. Between the two keV levels of 40 and 45 keV, there was no difference in the increase in diagnostic scans; however, at the level of 45 keV, the noise level is lower, even though this difference is not significant. All of the late arterial scans were deemed diagnostic, which means that there remain cases in which repetition is necessary. Hence, we propose generating an additional reconstruction at the keV level of 45 in all thoracoabdominal CTA at a PCD-CT. For clinical use, it is advantageous to reconstruct low-keV images instantaneously as the first series. This would help the responsible radiologist make a confident decision as to whether a repeat scan is necessary.

Virtual monoenergetic images have been in use since the introduction of dual-source CT scanners. The advantage of better contrast attenuation in low-energy VMIs on a EID-CT has been proven in many studies; however, this increase in attenuation comes with the disadvantage of a decrease in diagnostic confidence due to a higher noise level [23,24,25]. Leithner et al. compared optimized VMIs to standard VMI [26]. The study showed an improvement of diagnostic quality in lower keV levels due to noise-reducing reconstruction algorithms but still mentioned a significant rise in noise levels. In spite of the advantage of further noise reduction on a PCD-CT due to the ability to adjust the photon energy threshold [4,5], our study also showed a rise in noise level in the lower keV reconstructions. This concurs with other studies that pointed out an increase in noise, CNR, and SNR in lower keV reconstructions but have also proven a significant advantage compared to EID-CT scans [12,21]. This supports our subjective rating, where we were able to show that it is still possible to increase the number of diagnostic usable scans in case of decreased contrast with the low-keV reconstructions in spite of the significant increase in noise. We were able to show that even CT scans with low or very low attenuation can be improved, if not salvaged, by using VMI reconstructions with low-keV levels on a PCD-CT.

When looking at scans with regular attenuation, this implies that there is a possibility to reduce the amount of the contrast agent used. Many studies on EID-CT have shown that there is a potential to reduce the contrast used to about 50% when using lower keV reconstructions [27,28,29]. However, a possible increase in non-diagnostic images could be the result for TAVR patients with a decreased ejection fraction. Whether a previous measurement of the cardiovascular circulation time or a general increase in the delay between the contrast injection and the scan is the answer to this problem needs to be investigated. The noise level reduction in low-keV VMIs on a PCD-CT compared to a EID-CT has been shown in multiple studies and examined body parts [22,30]. In addition, the first studies have been published that combined an increase in attenuation of low-keV reconstructions with noise reduction on PCD-CT to reduce the amount of the iodine load. Emrich et al. proposed a reduction in contrast media concentration of up to 50% for coronary CTA due to their results on a phantom study [11]. However, clinical validation has yet to be conducted. However, Higashigaito et al. demonstrated the non-inferior image quality of PCD-CT reconstructions at 50 keV with a 25% reduction in contrast media volume compared to EID-CT [10]. It remains to be seen how the aspects of dose reduction and spectral imaging can and will be further combined in PCD-CT.

The main limitations of this study are the retrospective set up and that it was conducted in a single center. Furthermore, the subgroup analysis consisted partially of small patient groups. In particular, the very low contrast attenuation of subgroup 1 is fortunately uncommon, which is why we could not include more patients. However, further assessment in a prospective study is warranted to confirm those results and to investigate the possibility of contrast agent reduction.

In conclusion, low-keV VMI derived from PCD-CT datasets can be used to improve diagnostic image quality and even salvage thoracoabdominal CTA with suboptimal contrast attenuation. We propose 45 keV reconstructions to improve diagnostic confidence.

## Figures and Tables

**Table 1:** Basic demographic data.

Age, Years	77.4 ± 8.5
Sex (m/f)	40/15
Weight, kg	75.2 ± 10.8
Height, m	1.70 ± 0.07
BMI, kg/m²	25.9 ± 3.1
CTDIvol, mGy	4.1 ± 1
DLP, mGy·cm	473 ± 172.5

Data are mean ± standard deviation; BMI = body mass index, CTDIvol = CT dose index–volume, DLP = dose length product.

**Table 2:** Distribution of quantitative CT parameters per measured location.

keV	Loc	HU	Noise	CNR	SNR
40	ascending aorta	1338.8 ± 249.7	39.4 ± 9.7	34.2 ± 10.9	39.1 ± 13.4
40	descending aorta	1138.1 ± 242.7	51.1 ± 13.4	22.4 ± 7.7	33.2 ± 11.6
40	Infrarenal aorta	868.5 ± 232.1	48.8 ± 16.6	17.9 ± 7.3	25.1 ± 9.3
40	left CFA	414.9 ± 169.2	30.7 ± 9.8	12.4 ± 7.4	11.9 ± 5.8
40	right CFA	417.4 ± 172.7	32.6 ± 9.4	11.4 ± 6.2	12.0 ± 6.0
45	ascending aorta	1078.7 ± 223.1	34.2 ± 8.6	31.2 ± 9.9	34.7 ± 12.3
45	descending aorta	924.3 ± 200.8	45.1 ± 11.4	20.3 ± 7.1	29.7 ± 10.5
45	Infrarenal aorta	716.3 ± 175.1	43.0 ± 13.4	16.4 ± 6.4	22.9 ± 8.1
45	left CFA	349.7 ± 131.7	28.0 ± 8.5	11.2 ± 6.4	11.1 ± 5.1
45	right CFA	348.8 ± 135.8	29.2 ± 8.5	10.4 ± 5.5	11.1 ± 5.2
70	ascending aorta	456.2 ± 93.8	20.2 ± 4.7	20.8 ± 5.7	22.2 ± 7.1
70	descending aorta	388.3 ± 79.1	26.1 ± 6.5	13.6 ± 4.4	18.9 ± 5.9
70	Infrarenal aorta	305.0 ± 80.4	25.1 ± 7.8	10.8 ± 4.1	14.8 ± 5.0
70	left CFA	165.6 ± 53.7	17.3 ± 5.9	7.3 ± 4.2	8.0 ± 3.3
70	right CFA	167.3 ± 51.3	16.5 ± 5.6	7.8 ± 4.8	8.1 ± 3.1

Data are mean ± standard deviation; HU = Hounsfield Units, CNR = contrast-to-noise ratio, SNR = signal-to-noise ratio, CFA = common femoral artery.

**Table 3:** Number of diagnostic scans.

	keV 70	keV 40/45
Subgroup 1 *n* = 11	5 (45)	7 (64)
Subgroup 2 *n* = 22	17 (77)	20 (91)
Subgroup 3 *n* = 21	19 (90)	20 (95)
Subgroup 4 *n* = 41	38 (93)	40 (98)

Data are *n* (%).

## Data Availability

No new data were created or analyzed in this study. Data sharing is not applicable to this article.
